# Tocotrienols Attenuate White Adipose Tissue Accumulation and Improve Serum Cholesterol Concentration in High-Fat Diet-Treated Mice

**DOI:** 10.3390/molecules27072188

**Published:** 2022-03-28

**Authors:** Yugo Kato, Yoshinori Aoki, Chikako Kiyose, Koji Fukui

**Affiliations:** 1 Molecular Cell Biology Laboratory, Department of Functional Control Systems, Graduate School of Engineering and Science, Shibaura Institute of Technology, Fukasaku 307, Minuma-ku, Saitama 337-8570, Japan; nb19102@shibaura-it.ac.jp; 2 Mitsubishi Chemical Corporation, Marunouchi 1-1-1, Chiyoda-ku, Tokyo 100-8251, Japan; aoki.yoshinori.ma@m-chemical.co.jp; 3 Department of Nutrition and Life Science, Kanagawa Institute of Technology, Shimo-ogino 1030, Atsugi-shi 243-0292, Japan; kiyose@bio.kanagawa-it.ac.jp

**Keywords:** anti-obesity, tocotrienols, dyslipidemia, adipose tissue, neurotrophic factor

## Abstract

Tocotrienols (T3s), which are vitamin E homologs, have not only antioxidant function but also inhibitory effects on body weight gain and hepatic lipid droplet accumulation. However, the mechanisms of the anti-obesity effects of T3s are not yet understood. In this study, C57BL/6 mice were fed a high-fat diet in the presence or absence of T3s. Treatment with T3s inhibited white adipose tissue accumulation and elevation of serum cholesterol concentrations. Additionally, to clarify the relationship between obesity-induced cognitive dysfunction and the neuroprotective effect of T3s, cognitive function, brain oxidation, and protein expression levels of brain-derived neurotrophic factor (BDNF), which is strongly involved in neuronal growth and differentiation, were measured. Although mice behaviors were improved by oral T3 intake, there were no significant differences in brain oxidation levels and BDNF expression. These results suggest that T3s attenuate obesity via inhibition of body fat and serum cholesterol increase.

## 1. Introduction

Obesity induces several severe diseases, such as diabetes and cardiovascular disease, and over four million people die from obesity-related illnesses each year. The worldwide prevalence of overweight or obesity quadrupled from 1975 to 2016. Because this problem occurs not only in developed countries but also in developing countries, it has become a serious social problem worldwide. Obesity is recognized as a disease in many countries and is preventable via balanced nutrition (limitation of energy intake from fats and sugars, increase of fruit and vegetable consumption) and physical activity [1], but there are few drugs for treatment, so materials to treat obesity are highly sought after.

To find new materials with anti-obesity effects, understanding the mechanism of obesity is important. Increasing oxidation due to obesity may be one reason to raise the risk of developing various diseases [2,3]. Several kinds of evidence suggest that obesity also increases the risk of neurodegenerative diseases, such as Alzheimer’s disease (AD) and Parkinson’s disease [4,5]. In general, these diseases are strongly associated with the acceleration of brain oxidation [6]. If neurons are attacked by reactive oxygen species (ROS), they are gradually damaged and eventually die. Thus, obesity increases ROS production in living tissues and may induce cognitive dysfunction. However, there is still no direct evidence of obesity-induced brain oxidation. To find new substances that prevent obesity and severe secondary diseases caused by obesity, such as neurodegenerative diseases, the substance needs to have not only anti-obesity effects but also strong antioxidant functions.

Tocotrienols (T3s) are vitamin E (VE) homologs whose most notable biological function is as antioxidants [7,8]. It has been reported that T3s significantly suppress body weight gain in high-fat diet (HFD)-treated mice and inhibit liver lipid droplet accumulation [9,10,11]. However, the detailed mechanisms of these effects are still unclear. T3s play a role in the inhibition of 3-hydroxy-3-methylglutaryl-coenzyme (HMG-Co) A reductase, which catalyzes the conversion of HMG-CoA to mevalonic acid, and γ-T3 may arrest the cell cycle and induce apoptosis in 3T3-L1 adipocytes [12,13,14]. Cholesterol is made from mevalonic acid, and some HMG-CoA reductase inhibitors such as statins are prescribed for people with hypercholesterolemia [15,16]. However, there are few studies on the effects of T3s on animal cholesterol and white adipose tissue.

Neurotrophic factors, such as nerve growth factor (NGF) and brain-derived neurotrophic factor (BDNF), play an important role in the maintenance of cognitive function [17,18]. They are related to neuronal growth and differentiation, and serum BDNF levels are low in AD patients compared to normal healthy subjects [19]. Gene expressions are regulated by DNA methylation, miRNA, and histone modifications. It has been reported that BDNF mRNA transcription is regulated by Histone H3 acetylation [20].

In this study, to elucidate the anti-obesity effects of T3s, C57BL/6 mice were fed a HFD with T3s, and adipose tissue weights and serum cholesterol concentration of each mouse were measured. Additionally, cognitive function, brain oxidation levels, and BDNF protein levels of obese mice were assessed.

## 2. Results

### 2.1. T3s Attenuated Obesity via Inhibition of Adipose Tissue Accumulation and Lipid Metabolism Dysfunction

**Figure 1:**
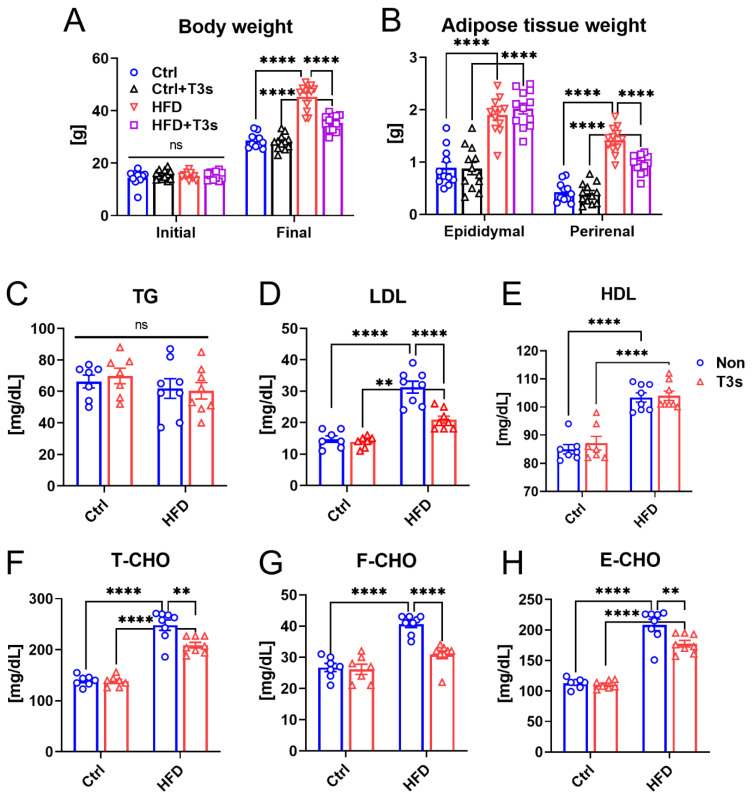
**T3s inhibited body weight gain and improved serum cholesterol levels.** (**A**) Body weights of each group on the first and final days of treatment. (Ctrl, *n* = 11; Ctrl + T3s, HFD and HFD + T3s, *n* = 12). (**B**) Weights of epididymal and perirenal fat. (Ctrl, *n* = 11; Ctrl + T3s, HFD and HFD + T3s, *n* = 12). (**C**–**H**) Serum cholesterol levels. (Ctrl and Ctrl + T3s, *n* = 7; HFD and HFD + T3s, *n* = 8). ** *p* < 0.01, **** *p* < 0.0001 two-way analysis of variance (ANOVA) with the Tukey Kramer method.

The body weights of all mice on the first and final day of the treatment period were measured (Figure 1A). Although no significant differences were observed on the initial day, treatment with HFD for 13 weeks significantly increased body weight compared to the control diet group (Ctrl). However, co-treatment with HFD and T3s (HFD + T3s) significantly inhibited body weight gain compared to the HFD-treated group. To clarify the change of white adipose tissue weight by T3s intake, the epididymal and perirenal fat were collected, and their weights were measured (Figure 1B). Although treatment with HFD significantly raised both fat weight, co-treatment with HFD and T3s significantly inhibited the perirenal fat accumulation compared to the HFD-treated group.

Treatment with HFD induced serum lipid metabolism dysfunction (Figure 1C–H). However, LDL, T-CHO, F-CHO, and E-CHO levels of the HFD + T3s group were improved compared to those of the HFD group. Interestingly, HDL levels were not significantly different in the presence or absence of T3s.

### 2.2. The Changes in Mice Behavior

**Figure 2:**
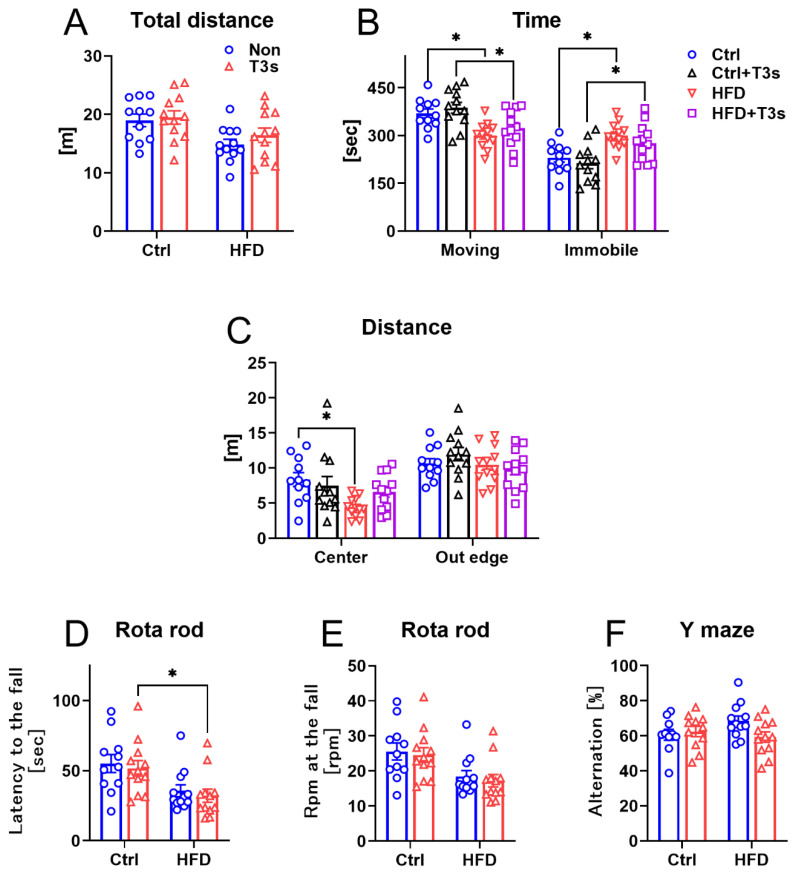
**The changes in mice behavior.** (**A**–**C**) Open field test. (Ctrl, *n* = 11; Ctrl + T3s, HFD and HFD + T3s, *n* = 12). (**D**,**E**) Latency to fall and rpm at fall in the rota rod test. (Ctrl, *n* = 11; Ctrl + T3s, HFD and HFD + T3s, *n* = 12). (**F**) Alternation score by Y-maze test. (Ctrl, *n* = 11; Ctrl + T3s, HFD and HFD + T3s, *n* = 12). * *p* < 0.05, two-way ANOVA with the Tukey–Kramer method.

Both HFD and HFD + T3s groups had low activity in the open field test (Figure 2A–C). Treatment with HFD and HFD + T3s significantly decreased total movement time and increased immobile time. Additionally, treatment with HFD significantly decreased the movement distance in the center of the open field apparatus, and the movement distance of HFD + T3s mice was not decreased compared to that of mice-treated control diet with T3s (Ctrl + T3s).

In the rota rod test, the time to fall from the rod of HFD + T3s mice was significantly faster than that of Ctrl + T3s mice (Figure 2D,E). However, no significant differences in the rpm at fall were observed.

There were no significant differences in the results of the Y-maze test (Figure 2F).

### 2.3. T3s Protected against HFD-Induced Hepatic Damage

**Figure 3:**
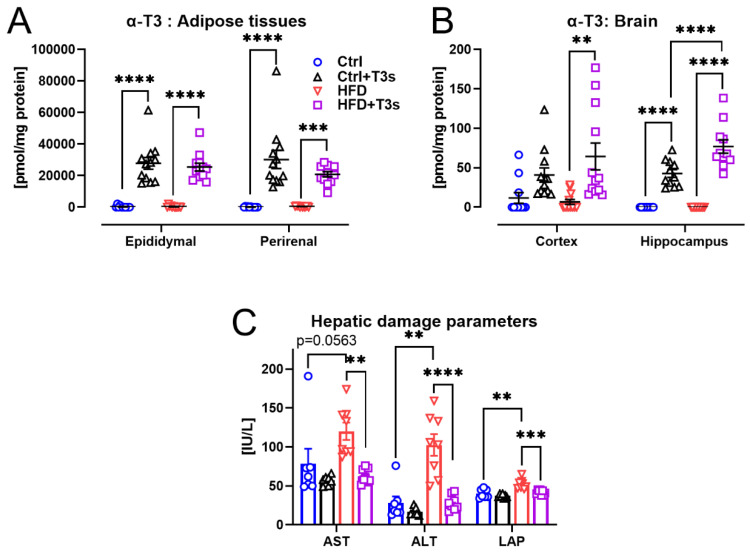
**α-T3 levels and serum hepatic parameters.** (**A**) α-T3 levels in adipose tissues. (Ctrl, *n* = 11; Ctrl + T3s, HFD and HFD + T3s, *n* = 12). (**B**) α-T3 levels in each brain region. (Cortex: Ctrl, *n* = 11; Ctrl + T3s, HFD and HFD + T3s, *n* = 12; Hippo.: Ctrl, Ctrl + T3s, HFD and HFD + T3s, *n* = 11). (**C**) Index of hepatic damage in serum. (Ctrl and Ctrl + T3s, *n* = 7; HFD and HFD + T3s, *n* = 8). ** *p* < 0.01, *** *p* < 0.001, **** *p* < 0.0001, two-way ANOVA with the Tukey–Kramer method.

T3 levels in each tissue and the hepatic damage indexes in serum were measured. Co-treatment with T3s significantly increased tissue T3 levels (Figure 3A,B).

The levels of ALT and LAP in serum were significantly elevated by HFD-induced obesity. However, co-treatment with HFD and T3s prevented their increase (Figure 3C).

### 2.4. HFD and T3s Did Not Alter Brain Oxidation Levels

**Figure 4:**
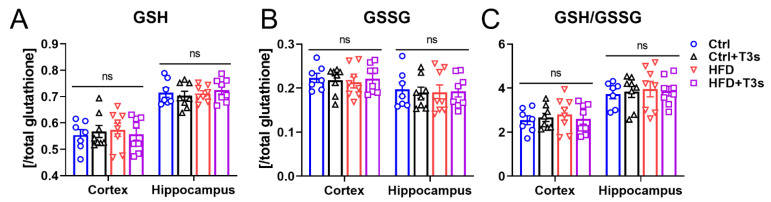
**Brain oxidation levels.** (**A**,**B**) GSH and GSSG levels in each mouse brain. (Ctrl, *n* = 7; Ctrl + T3s, HFD and HFD + T3s, *n* = 8). (**C**) Ratio of GSH and GSSG. (Ctrl, *n* = 7; Ctrl + T3s, HFD and HFD + T3s, *n* = 8).

The redox balance of glutathione was measured as an indicator of brain oxidation levels (Figure 4). As mouse movements in the open field test were significantly changed by treatment with HFD or T3s, it was possible that the brain oxidation levels would be altered by HFD or T3s. However, GSSG was not increased in HFD mouse brains. Additionally, the ratio of GSH and GSSG was not changed.

### 2.5. HFD and T3s Did Not Influence the Expression of BDNF

**Figure 5:**
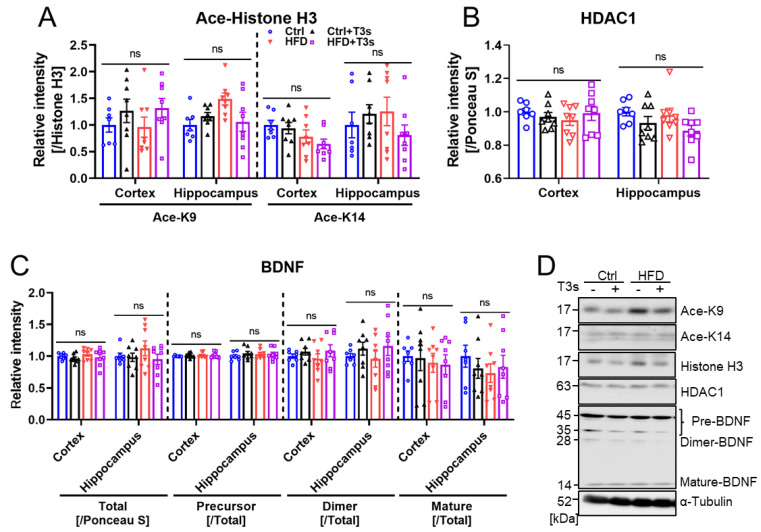
**Histone H3 acetylation and BDNF in the brain.** (**A**–**C**) Quantitative results of acetylated (Ace)-histone H3 (K9), (K14), HDAC1, total-BDNF, precursor-BDNF (Pre-), dimer-BDNF (Dimer-), and mature-BDNF. (**D**) Representative western blotting images. (Ctrl, *n* = 7; Ctrl + T3s, HFD and HFD + T3s, *n* = 8).

Treatment with HFD significantly altered mouse movement in the open field test. The expressions of histone H3 acetylation and BDNF in the brains were assessed using western blotting (Figure 5). No significant differences were observed in any parameter. However, dimer-BDNF expression tended to increase (but not significantly) by treatment with T3s.

## 3. Discussion

As obesity raises the risk of several diseases, such as type-2 diabetes, cardiovascular disease, and atherosclerosis, the prevention of obesity is very important [21,22,23]. It has been reported that T3s have an anti-obesity effect in HFD-treated mice [9,10,11]. However, the underlying mechanism of this effect has been unclear. In the present study, treatment with HFD increased body weight, and co-treatment with T3s significantly inhibited body weight gain. It has been reported that γ-T3 arrests the cell cycle and leaks cytochrome C from the mitochondria to the cytosol in 3T3-L1 adipocytes. As the results of cytochrome C release and caspase-3 activation, apoptosis is induced [12]. To clarify the effects of T3s on white adipocyte in vivo, the epididymal and perirenal adipose tissue weights were measured. Treatment with T3s significantly inhibited perirenal fat accumulation in HFD-treated mice. However, epididymal fat weight was not changed by T3s treatment. It may be hard to reduce epididymal fat weight compared to perirenal fat due to the need to protect the testes, which are needed for reproduction. T3s did not modulate the final body weight in control mice. This result indicated that T3s did not negatively affect normal mouse growth. Although treatment with T3s inhibited the body weight gain in obese mice compared to the untreated group, the final body weight of the T3s-treated obese mice was still higher than that of the T3s-treated control mice. The inhibitory effect of body weight gain of T3s did not completely arrest the HFD-induced body weight gain. While it is certain that T3s inhibit HFD-induced body weight gain, how adipose tissue weight is decreased by T3s remains unclear. Further study is needed to resolve this question.

Previously, it was reported that T3s inhibit HFD-induced hepatic lipid droplet accumulation [11]. To reveal the effects of T3s on serum lipid metabolism, cholesterol concentrations in serum were measured. Treatment with HFD caused an increase in serum cholesterol levels, and T3s significantly decreased these parameters. HMG-CoA reductase plays an important role in cholesterol synthesis, and some HMG-CoA reductase inhibitors are prescribed for people with dyslipidemia [13,14,15,16]. It has also been reported that T3s have an inhibitory effect against HMG-CoA reductase [13]. Additionally, T3s induce the ubiquitination and degradation of HMG-CoA reductase [24], so it is possible that T3s may improve serum cholesterol profiles via HMG-CoA reductase inhibition. Therefore, the previous results of hepatic lipid droplet reduction by T3s may be mediated by their serum cholesterol-lowering effect.

The index of obesity, such as body weight and serum cholesterol concentration, is related to the brain BDNF. Rios et al. generated conditional mutants in which BDNF is eliminated from the brain, and the mutants had a drastic increase in body weight and elevated serum parameters, including cholesterol [25]. In our study condition, while body weight and serum cholesterols were increased, there was no significant difference in brain BDNF levels. Our results indicate that there is no relationship between HFD-induced obesity development and brain BDNF levels.

Mouse behavior was assessed using an open field apparatus. Treatment with HFD decreased total distance moved in the center of the open field apparatus, similar to the effects of depression and anxiety-like behavior, but no significant difference was found in this parameter between Ctrl + T3s and HFD + T3s mice. People with depression exhibit low serum BDNF levels [26]. Additionally, it has been reported that hippocampal BDNF levels of anti-depressive agent-treated patients are significantly higher than those of non-treated subjects [27]. To clarify the reasons why HFD or T3s influenced mouse movement, the expressions of BDNF and acetylated histones, which are related to BDNF transcription, were measured [28,29]. However, there were no significant differences in these expressions. Although relationships between depression, oxidative stress, and inflammation have been reported, there were no significant differences in brain oxidation levels in this study [30,31,32,33]. Inflammation is accelerated in obese bodies [34,35], so treatment with HFD may alter mouse behavior via changes in inflammation levels. While it was revealed that HFD modulated mouse behavior in this study, the mechanisms remain unknown.

In this study, the coordination ability as an index of cognitive function of mice was assessed using rota rod test. The fall latency from the rod of HFD + T3s mice in the rota rod test was significantly decreased compared to that of the Ctrl + T3s mice. It is possible that this result was influenced by mouse body weight (because the body weight of each mouse group was significantly different, the rota rod test may not be suitable for the assessment of obese mouse cognition). Treatment with HFD or T3s did not change the cognition in our experimental conditions (diet, treatment duration, and cognitive apparatus). We may need to change our experimental scheme in future experiments.

Understandably, treatment with T3s significantly increased tissue T3 levels. It was previously reported that HFD accumulates lipid droplets in the liver and that T3s inhibit it [11]. In this study, some serum hepatic damage parameters were measured. Diet-induced obesity increased hepatic damage, and T3s provided a protective effect. One potential cause of hepatic damage from obesity is oxidative damage [36,37]. It is well known that T3s have a strong antioxidant function, and T3s certainly reach the liver by oral intake [11]. Thus, HFD-induced hepatic damage may have been prevented by the antioxidant function of T3s.

There were no significant differences in Western blotting of the examined proteins. BDNF is strongly related to cognition, but in this study, cognitive function did not change, so BDNF expression did not change either [18]. There are some isoforms of BDNF, and some researchers have focused on mature-BDNF (14 kDa) to investigate the relationships between cognition and BDNF function [38,39,40,41,42]. However, the dimer (28 kDa) and precursor forms of BDNF (35 and 45 kDa) were also detected under our experimental conditions. The dimer-BDNF promotes neural survival and growth by binding to its receptor (tropomyosin receptor kinase B (TrkB)) [43]. In contrast, the binding between precursor-BDNF and low-affinity nerve growth factor receptor (p75NTR) leads to apoptosis and is related to long-term depression [42,43]. For these reasons, not only the expression of mature-BDNF, but also that of other forms must be considered. However, there were no significant differences in any of the isoforms. Additionally, BDNF mRNA is transcribed after histone acetylation, and histones are deacetylated by HDAC [44,45]. T3s are changed to carboxyhydrochroman derivatives after ω-hydroxylation and β-oxidation. The carboxyhydrochroman derivatives may have HDAC inhibitory function [44,45]. Given this, we expected that T3s would upregulate histone acetylation levels via HDAC inhibition. However, there were no significant differences in acetylated-histone or HDAC1 expression and no significant change in BDNF expression.

## 4. Materials and Methods

### 4.1. Animals

All animal experiments were approved by the Animal Protection and Ethics Committee of Shibaura Institute of Technology (Approval number #20002). Four-week-old C57BL/6 Ncr male mice were obtained from Japan SLC, Inc. (Hamamatsu, Japan). Three or four mice were housed in each cage for 13 weeks under a 12 h light/dark cycle in a controlled-temperature room (22 ± 2 °C). All mice could freely access food and water. The mice were fed with either a HFD (#D12492, Research Diets Inc., New Brunswick, NJ, USA) or a control diet (Ctrl, #D12450, Research Diets Inc.) in the presence (Ctrl + T3s, HFD + T3s) or absence of T3s (50 mg/100 g diet; α-:β-:γ-:δ- = 33.4:4.4:46.7:15.2, Mitsubishi Chemical Foods Corp., Tokyo, Japan). The nutrient composition of each diet was described in Supplementary Materials Table S1. After 13 weeks, the serum, epididymal and perirenal fat, cerebral cortex (Cortex), and hippocampus were collected for each assessment. The weights of each fat were measured as soon as possible after collection from mouse bodies using an electronic scale.

### 4.2. Behavioral Assessment

#### 4.2.1. Open Field Test

The mice were placed in the apparatus (40 × 40 cm) and allowed to move freely for 10 min (min). The total movement distance and time, immobile time, and the movement distances in the center or outer edge were analyzed using ANY-maze software (Stoelting Co., Wood Dale, IL, USA).

#### 4.2.2. Rota Rod Test

The rota rod test (Muromachi Kikai Co., Ltd., Tokyo, Japan) was used to assess coordinated movement ability as described previously with some modifications [46]. The rod was accelerated from 5 to 50 rpm over a duration of 120 s. The latency to fall and the rpm at the fall were measured.

#### 4.2.3. Y-Maze Test

A Y-maze apparatus (Muromachi Kikai Co., Ltd.) was used to measure the short-term memory of each mouse, as described previously with some modifications [11]. The mice moved freely in the apparatus for 10 min, and the alternation score was analyzed using ANY-maze software (Stoelting Co.).

### 4.3. VE Measurement

VE volume in all tissues was measured using high-performance liquid chromatography (HPLC), as described previously with some modifications [11]. VE was extracted by the saponification extraction method. 2,2,5,7,8-pentamethyl 6-chromanol (PMC) (Sigma Aldrich Corp., St Louis, MO, USA) was used as the internal standard. After saponification, VE in the samples was extracted using a mixture of hexane and ethyl acetate and enriched into HPLC-grade methanol. VE in this solution was measured using HPLC with electrochemical detection (Shiseido Co., Ltd., Tokyo, Japan). The mobile phase was composed of H_2_O and HPLC-grade methanol (3:97) including 50 mM of NaClO_4_·H_2_O. The HPLC column (Develosil C30-UG-3 (2.0 × 250 mm; Nomura Chemical Co., Ltd., Aichi, Japan)) was used at 5 °C.

### 4.4. Reduced (GSH) and Oxidized (GSSG) Glutathione Concentrations

The concentrations of GSH and total glutathione were measured using a GSH/GSSG Ratio Detection Assay Kit II; (#ab205811, Abcam Plc., Cambridge, UK). The concentration of GSSG was calculated by subtracting GSH from the total and dividing by 2. FLUOROSKAN (Thermo Fisher Scientific Inc., Waltham, MA) was used for measuring fluorescence.

### 4.5. Serum Parameters

Triglyceride (TG), low-density lipoprotein (LDL), high-density lipoprotein (HDL), total-cholesterol (T-CHO), free-cholesterol (F-CHO), esterified-cholesterol (E-CHO), aspartate aminotransferase (AST), alanine aminotransferase (ALT), and leucine transpeptidase (LAP) were measured by an animal inspection service (Oriental Yeast Co., Ltd., Tokyo, Japan).

### 4.6. Western Blotting

Western blotting was performed as described previously with some modifications [47]. Twenty micrograms of sample protein were loaded onto 12% sodium lauryl sulfate-polyacrylamide gels. The protein on the gels was transferred to nitrocellulose membranes. The membranes were stained using Ponceau S staining solution (Sigma Aldrich Corp.). Two percent skim milk or bovine serum albumin solution was used as the blocking buffer and incubated with membranes for 1 h at room temperature. Then membranes were reacted with anti-HDAC1 (#34589, 1:2000 dilution, Cell Signaling Technology (CST) Inc., Danvers, MA, USA), anti-Histone H3 (#4499, 1:4000 dilution, CST Inc.), anti-acetyl-Histone H3 (K9) (#9649, 1:1000 dilution, CST Inc.), anti-acetyl-Histone H3 (K14) (#7627, 1:500 dilution, CST Inc.), anti-BDNF (#ab108319, 1:2500 dilution, Abcam Plc.) and anti-α-Tubulin (#2125, 1:4000 dilution, CST Inc.) overnight at 4 °C. Anti-rabbit IgG HRP antibody (Promega Corp., Madison, WI, USA) was used as the secondary antibody at 1:4000 dilution. The chemiluminescent signals were detected by a LAS-3000 (FUJIFILM Corporation, Tokyo, Japan). The relative intensities were measured using Image Quant software (Cytiva, Tokyo, Japan).

### 4.7. Statistical Analysis

All data are presented in all figures as means ± SE and were analyzed by two-way ANOVA with the Tukey–Kramer method using GraphPad Prism 9 (Graph Pad Software, San Diego, CA, USA). *p* < 0.05 was considered statistically significant.

## 5. Conclusions

In conclusion, T3s exerted anti-obesity effects such as the attenuation of body weight gain, serum cholesterol elevation, and white adipose tissue accumulation in high-fat diet-treated mice. Additionally, T3s also inhibited hepatic damage from obesity. However, while it has been reported that obesity induces cognitive dysfunction, no change was detected in this study. Further study is needed to understand the changes and relationship between brain function and brain oxidation in obese mice.

## Data Availability

All data generated or analyzed during this study are included in this published article.

## Supplementary Materials

The following supporting information can be downloaded at: https://www.mdpi.com/article/10.3390/molecules27072188/s1, Table S1: Diet compositions.

## Sample Availability

Samples of the compounds are available from the authors.
